# Development of Chloroplast Microsatellite Markers and Evaluation of Genetic Diversity and Population Structure of Cutleaf Groundcherry (*Physalis angulata* L.) in China

**DOI:** 10.3390/plants12091755

**Published:** 2023-04-25

**Authors:** Shangguo Feng, Kaili Jiao, Zhenhao Zhang, Sai Yang, Yadi Gao, Yanyun Jin, Chenjia Shen, Jiangjie Lu, Xiaori Zhan, Huizhong Wang

**Affiliations:** 1 College of Life and Environmental Science, Hangzhou Normal University, Hangzhou 311121, China; 2 Zhejiang Provincial Key Laboratory for Genetic Improvement and Quality Control of Medicinal Plants, Hangzhou Normal University, Hangzhou 311121, China; 3 Orient Science & Technology College, Hunan Agricultural University, Changsha 410128, China

**Keywords:** *Physalis angulata*, Solanaceae, simple sequence repeat, genetic variation, population structure

## Abstract

Cutleaf groundcherry (*Physalis angulata* L.), an annual plant containing a variety of active ingredients, has great medicinal value. However, studies on the genetic diversity and population structure of *P. angulata* are limited. In this study, we developed chloroplast microsatellite (cpSSR) markers and applied them to evaluate the genetic diversity and population structure of *P. angulata*. A total of 57 cpSSRs were identified from the chloroplast genome of *P. angulata*. Among all cpSSR loci, mononucleotide markers were the most abundant (68.24%), followed by tetranucleotide (12.28%), dinucleotide (10.53%), and trinucleotide (8.77%) markers. In total, 30 newly developed cpSSR markers with rich polymorphism and good stability were selected for further genetic diversity and population structure analyses. These cpSSRs amplified a total of 156 alleles, 132 (84.62%) of which were polymorphic. The percentage of polymorphic alleles and the average polymorphic information content (PIC) value of the cpSSRs were 81.29% and 0.830, respectively. Population genetic diversity analysis indicated that the average observed number of alleles (*Na*), number of effective alleles (*He*), Nei’s gene diversity (*h*), and Shannon information indices (*I*) of 16 *P. angulata* populations were 1.3161, 1.1754, 0.1023, and 0.1538, respectively. Moreover, unweighted group arithmetic mean, neighbor-joining, principal coordinate, and STRUCTURE analyses indicated that 203 *P. angulata* individuals from 16 populations were grouped into four clusters. A molecular variance analysis (AMOVA) illustrated the considerable genetic variation among populations, while the gene flow (*Nm*) value (0.2324) indicated a low level of gene flow among populations. Our study not only provided a batch of efficient genetic markers for research on *P. angulata* but also laid an important foundation for the protection and genetic breeding of *P. angulata* resources.

## 1. Introduction

Cutleaf groundcherry (*Physalis angulata* L.) is an important annual herbaceous plant from the Solanaceae family, mainly distributed in China, Japan, India, Australia, and the Americas [1,2]. *P. angulata* has high potential medicinal value and a very long history of being used in traditional medicines around the world. Recent phytochemical and pharmacological studies have confirmed that *P. angulata* is rich in vitamins, minerals, antioxidants, and many important pharmacologically active constituents, including antibacterial, anti-inflammatory, and anticancer ingredients [3,4,5,6]. In many countries, such as China, Indonesia, Peru, Mexico, and Brazil, *P. angulata* is often used to treat a variety of illnesses, including dermatitis, tracheitis, impaludism, rheumatism, hepatitis, and analogous conditions [7,8,9,10]. Recently, increasing attention has been paid to the phytochemical and pharmacological aspects of *P. angulata*, and a variety of bioactive steroids with antitumor activities, including physagulins (A−Q), physangulidines (A−C), withangulatins (A−I), physalins (B, D, F−H), and withaminimin, have been isolated from the species [4,5,11]. Due to their important medicinal value, *P. angulata* plants have been widely cultivated in some regions of China for decades.

Genetic research to determine genetic diversity and population dynamics can be invaluable when forming and revising a species management plan, as maintaining diversity is critical for conservation [12,13,14]. Information about genetic structure is essential to understanding the scales over which dispersal, genetic drift, and selection operate in populations [15]. The design of microsatellite (simple sequence repeat, SSR) markers is based on conserved nucleotide sequences on both sides of simple repeat sequences, and the polymorphism among alleles is reflected by detecting the difference in the number of repeats [16]. SSRs are widely distributed in the nuclear and organellar genomes of eukaryotes [17,18,19,20]. SSR markers are considered to be among the most ideal molecular markers for the study of plant genetics [14,18]. Chloroplast microsatellites (cpSSRs) are widely distributed in the chloroplast genomes of plants. CpSSR markers not only have the advantages of co-dominance, high polymorphism, and multi-allelic loci, but also have the characteristics of a slow evolution rate, low molecular weight, relative conservation, simple structure, and single-parent inheritance [21]. In recent years, cpSSR markers have been widely used in plant phylogeography [22], species identification [21], phylogeny [23], and genetic diversity and population structure studies [24].

To date, several molecular marker techniques, including the use of SSRs, ISSRs, InDels, and SNP markers, have been used to analyze the genetic diversity of some *Physalis* species, including *P. peruviana*, *P. philadelphica*, and *P. floridana* [25,26,27,28]. Until recently, however, no cpSSR markers have been developed within the genus Physalis, and there are no reports of cpSSR marker usage for *P. angulata*. Moreover, there have been no published molecular marker-based studies of genetic diversity in *P. angulata* populations with a large number of samples. Although *P. angulata* has many important applications, researchers and plant breeders have not given enough attention to its conservation and improvement. Consequently, the production, utilization, and improvement of *P. angulata* are severely restricted.

Hence, this study was initiated with the aim of developing cpSSR markers to evaluate the genetic diversity and population structure in *P. angulata* germplasm collections in China. The findings can eventually serve as an important basis for the genetic improvement and sustainable conservation of *P. angulata* resources.

## 2. Results

### 2.1. Characterization of the Developed cpSSR Markers

**Figure 1:**
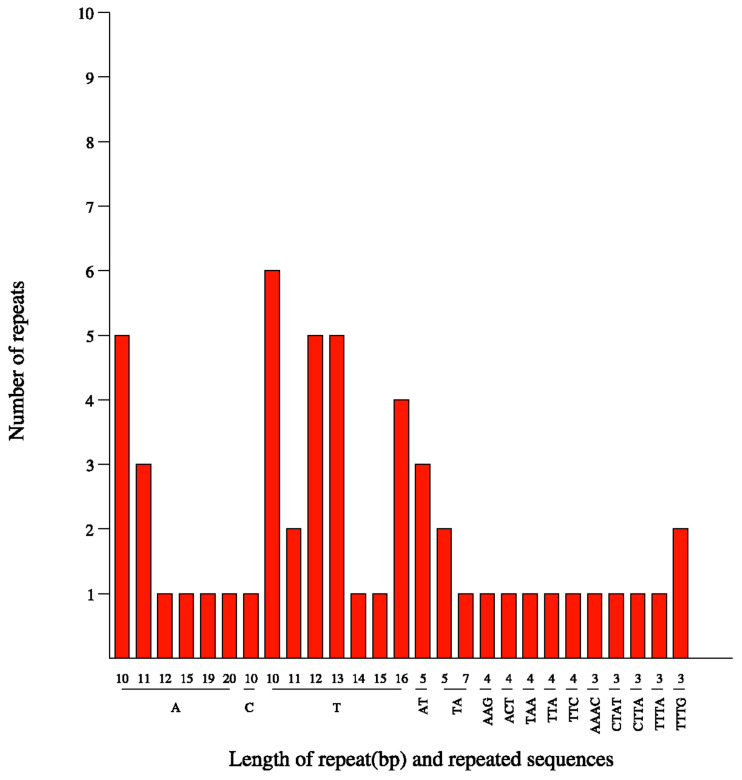
Types of SSR motifs detected in the *P. angulata* chloroplast genome.

In total, 57 cpSSR motifs (including three complex SSR types) were detected in the chloroplast genome of *P. angulata*, most of which were single-base repetitions dominated by A and T (Table 1 and Table 2). Information about the 57 SSR motifs is shown in Table S1. Among all detected SSR loci, mononucleotides were the most abundant, with 39 loci (68.24% of the total). This was followed by tetranucleotides, with seven loci (12.28% of the total), dinucleotides, with six loci (10.53% of the total), and trinucleotides, with five loci (8.77% of the total) (Table 1). Of the mononucleotide motifs, T (24 of 39, 61.54%) was the most abundant, followed by A (14 out of 39, 35.90%). The C motif was the least frequently observed (one of 39, 0.26%) mononucleotide. The dinucleotide motifs were mainly AT and TA, each accounting for 50%, respectively. Trinucleotide motifs included AAG, ACT, TAA, TTA, and TTC, accounting for 20% each, respectively. Of the tetranucleotide motifs, TTTA (28.57%) and TTTG (28.57%) were the most abundant, followed by AAAC (14.29%), CTAT (14.29%), and CTTA (14.29%) (Table 2 and Figure 1). The average lengths (bp) of the mononucleotide, dinucleotide, trinucleotide, and tetranucleotide cpSSRs were 12.04, 10.67, 11.6 and 12, respectively (Table S1). Additionally, 39 SSR motifs (68.42%) had repeat numbers greater than or equal to 10 (Table 2 and Figure 1).

In this study, 54 cpSSR primer pairs were developed and designed on the basis of the 57 cpSSR motifs detected in the *P. angulata* chloroplast genome using Primer Premier 5 with manual correction (Table S2). The amplification segments of these cpSSR primer pairs were distributed in four regions (LSC, SSC, IRA, and IRB) of the *P. angulata* chloroplast genome. Among them, 41 were distributed in the LSC region, along with five in the SSC region, four in the IRA region, and four in the IRB region. Through screening the 54 cpSSR primer pairs with six *P. angulata* genomic DNA samples, a total of 30 cpSSR primer pairs (55.6%) with good stability, high polymorphism, and clear electrophoresis bands were chosen for further genetic diversity analyses of the *P. angulata* populations (Table 3).

### 2.2. CpSSR Analysis

A total of 156 alleles were amplified by the 30 cpSSR primer pairs, with a range of two (KzcpSSR19, KzcpSSR21, KzcpSSR25, KzcpSSR37, KzcpSSR38, KzcpSSR39, and KzcpSSR53) to 12 (KzcpSSR04), and an average of 5.2 bands per primer pair (Table 3). The actual lengths of the amplified products ranged from 100 to 300 bp. Overall, 132 of the 156 amplified alleles were polymorphic, accounting for 84.62%, with an average of 4.4 alleles per pair of primers. The percentage of polymorphic alleles across the primer pairs ranged from 25.0% to 100.0%, with an average of 81.29%. The PIC value of each primer pair ranged from 0.550 to 0.945, with an average of 0.830 (PIC > 0.500). This indicates that these alleles contain abundant genetic information and can be used to study the genetic diversity and genetic structure of *P. angulata* populations.

### 2.3. Genetic Diversity Analysis

The genetic diversity of the 16 *P. angulata* populations (n = 203) was evaluated, revealing high mean per population estimates of allele and genetic diversity (*Na* = 1.3161, *Ne* = 1.1754, *h* = 0.1023, and *I* = 0.1538; see Table 4). The *PL* of the 16 *P. angulata* populations ranged from 10 (DQ) to 85 (YN and XS), with an average of 49, while the *PPL* ranged from 6.41% (DQ) to 54.49% (YN and XS), with an average of 31.61%. The *Na* per population ranged from 1.0641 (DQ) to 1.5449 (XS and YN), with an average of 1.3161. The *Ne* across the 16 populations ranged from 1.0371 (DQ) to 1.2984 (XS), with a mean of 1.1754. The *I* per population ranged from 0.0325 (DQ) to 0.2640 (XS), with an average of 0.1538. At the population level, the *PPL*, *Na*, *Ne*, and *I* values were the highest in the XS population, indicating that this population had the highest level of genetic diversity. The 16 populations were ranked by genetic diversity as follows: XS > YN > WZ > XJ > PJ > HG > JJ > NJZ > YW > TZ > LA > NH > LH > NJX > HZ > DQ.

### 2.4. Genetic Differentiation and Gene Flow

Nei’s genetic distance, calculated on the basis of a pairwise comparison, ranged from 0.0329 (between DQ and YW) to 0.8268 (between NH and NJX), with an average of 0.3401. The majority of Nei’s genetic identity ranged from 0.4374 (between NH and NJX) to 0.9677 (between TZ and LH), with an average of 0.7386 (Table 5). A Mantel test conducted to assess the correlation between genetic distance and geographical distance among the populations of *P. angulata* revealed no significant correlation (*r* = −0.176, *p* = 0.055).

At the species level, the *Ht* of the *P. angulata* population was 0.3224, the *Hs* was 0.1023, and the *Gst* was 0.6827 (Table 6). The results indicated that 68.27% of the genetic variation occurred between *P. angulata* populations, and 31.73% of the genetic variation occurred within *P. angulata* populations. The *Nm* of *P. angulata* populations was 0.2324, indicating that there was a low level of gene exchange between them. Furthermore, an AMOVA was performed to assess the molecular variations among *P. angulata* populations (Table 7). The results of the AMOVA revealed that 29% of total molecular variations were contributed by differences within populations, while the remaining 71% of total molecular variations were due to differences among populations. Population differentiation (PhiPT = 0.706) was significant (*p* = 0.001), which was consistent with the results from the analysis of Nei’s genetic diversity, indicating that the genetic differentiation between populations was higher than that within populations.

### 2.5. Genetic Relationships

**Figure 2:**
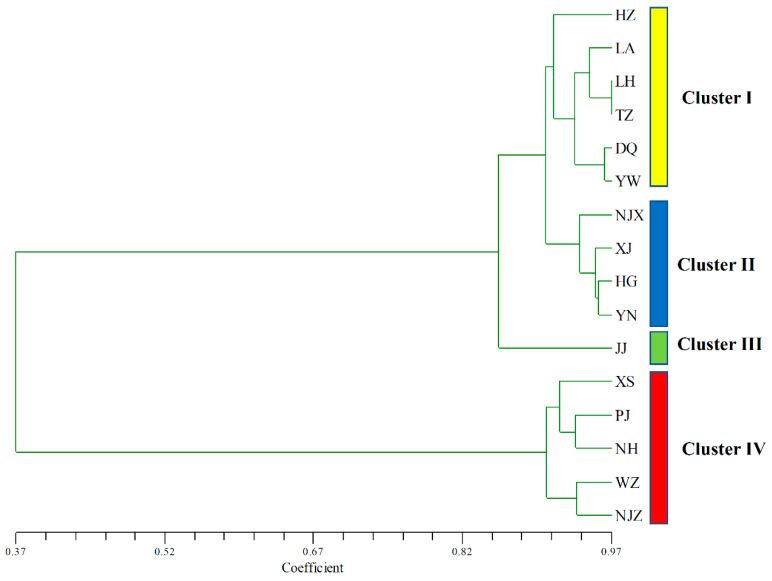
Dendrogram of 16 populations of *P. angulata* based on UPGMA analysis.

The UPGMA dendrogram of *P. angulata* populations was constructed on the basis of Nei’s genetic distance, which is an accurate reflection of the genetic relationships among populations. The UPGMA tree showed that the 16 populations could be divided into four major clusters (Clusters I, II, III, and IV) (Figure 2). Cluster I contained six populations, namely, HZ, LA, LH, TZ, DQ, and YW. Cluster II included four populations, namely, NJX, XJ, HG, and YN, while population JJ formed a separate cluster (Cluster III). The XS, PJ, NH, WZ, and NJZ populations were separated from the others and collectively grouped into Cluster IV.

**Figure 3:**
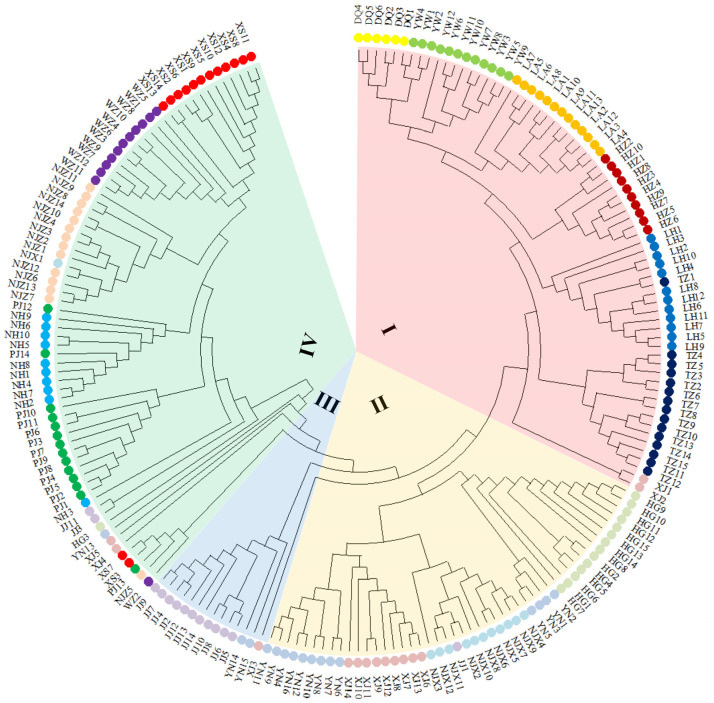
Neighbor-joining (NJ) phylogenetic tree of 203 individuals from 16 *P. angulata* populations. All tested samples were grouped into four clusters: I, II, III and IV.

To verify the results of the UPGMA analysis, an NJ tree of the 203 individuals was constructed on the basis of genetic distance (Figure 3). The results of the NJ tree analysis showed that the 203 *P. angulata* individuals were grouped into four distinct clusters, consistent with the results of the UPGMA analysis. The findings indicated that most of the individuals from the same population clustered together, but there were also a few individuals from the same population who grouped separately into different clusters. For example, almost all individuals in the XJ population were grouped into Cluster I, and the remaining individuals were grouped into Cluster II and Cluster III (Figure 3). Similar results were also observed in the JJ, YN, and NJX populations.

**Figure 4:**
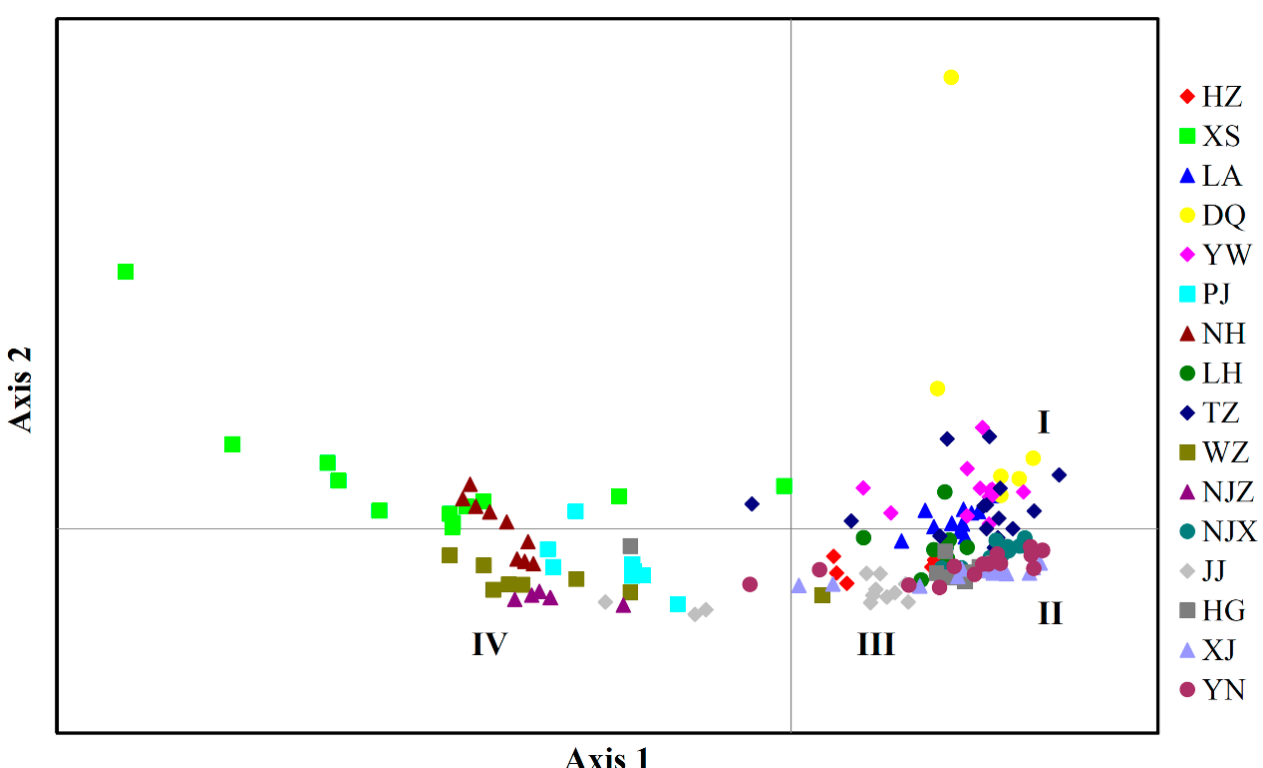
Principal coordinate analysis (PCoA) of 203 individuals of *P. angulata* based on GenAlEx analysis. All tested samples were grouped into four clusters: I, II, III and IV.

**Figure 5:**
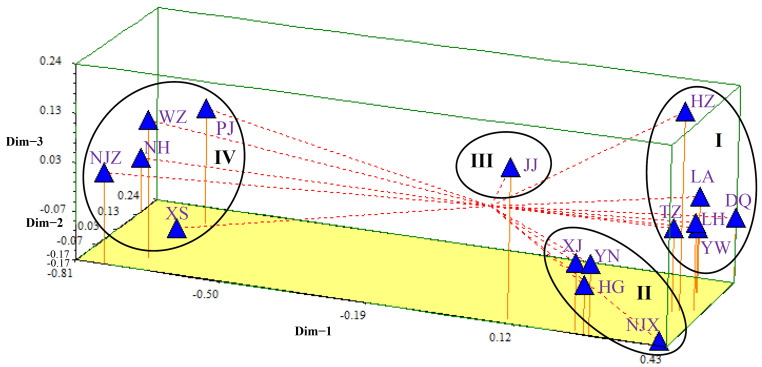
Principal coordinate analysis (PCoA) of 16 populations of *P. angulata* based on NTSYS analysis. All tested samples were grouped into four clusters: I, II, III and IV.

PCoAs, based on the genetic distance matrix, were then performed to more intuitively understand the genetic relationship among the 203 samples and 16 populations of *P. angulata* (Figure 4 and Figure 5). The percentage variance among the 203 samples attributable to the first two principal coordinate axes explained 35.04% and 14.55% of the molecular variance (Figure 4). Meanwhile, the percentage variance among the 16 populations attributable to the first three principal coordinate axes explained 76.67%, 5.95%, and 3.26% of the total molecular variance (Figure 5). Interestingly, the results of the two-dimensional PCoA among 203 samples and three-dimensional PCoA among 16 populations were consistent with the results of the UPGMA and NJ clustered tree analysis (Figure 2 and Figure 3). All the samples of *P. angulata* from 16 populations were classified into four groups.

### 2.6. Population Structure

**Figure 6:**
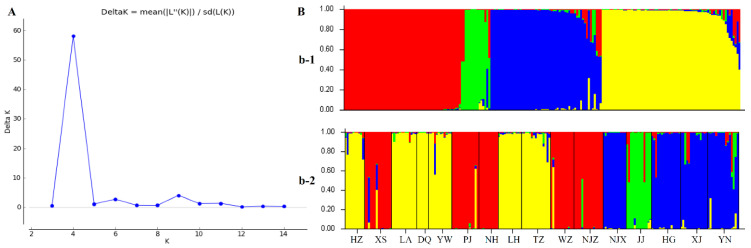
Population genetic structure. (**A**) Estimation of the best subpopulation numbers using delta K values for K ranging from 2 to 15. (**B**) Genetic structural plot of 16 *P. angulata* populations based on structure analysis. (b-1) K = 4, sorted by Q via STRUCTURE. (b-2) K = 4, samples displayed in order of *P. angulata* populations.

Population structure analysis was employed to rebuild the genetic relationship among the 16 *P. angulata* populations using the newly developed cpSSR markers. The results of the Structure Harvester analysis showed that the most likely value of K in the Bayesian clustering analysis was four (Figure 6A), indicating the presence of four main groups within the 16 *P. angulata* populations. The results of the Bayesian clustering analysis using STRUCTURE software (Figure 6B) confirmed the results obtained from the UPGMA dendrogram, NJ tree, and PCoA. The first cluster (yellow color) contained the HZ, LA, DQ, YW, LH, and TZ populations. The second cluster (red color) consisted of the XS, PJ, NH, WZ, and NJZ populations. The JJ population alone was placed into the third cluster (green color), while the NJX, HG, XJ, and YN populations were placed into the fourth cluster (blue color). Samples from the same population tended to be concentrated together, indicating that the genetic relationship between samples within a population was closer than the genetic relationship between populations. However, the results also showed that some individuals were crossed and overlapped among the populations, which indicates that there was some gene exchange and mutual penetration among the populations.

## 3. Discussion

SSR markers are popular tools for use in DNA marker technology and are extensively applied to the analysis of genetic diversity in plant populations. As a type of SSR marker, cpSSRs have the characteristics of high mutability and high conservation of chloroplast genome sequences. CpSSR markers are simple, efficient, and easy to operate, and they can reveal a high level of population diversity. CpSSRs have been used successfully to reveal genetic diversity among many plants [29,30,31,32]. However, to date, there have been no studies related to *P. angulata* SSR development, and no DNA marker technique has been employed to analyze the genetic diversity among *P. angulata* populations. In the present study, we developed a batch of SSR markers and used them to study the genetic diversity among *P. angulata* populations.

Mononucleotides (68.24% of the total) were the most common SSR loci detected in our study, followed by tetranucleotides (12.28% of the total). These proportions are similar to those of cpSSR marker types found in many plants [33,34,35]. Polymorphism is an important index for evaluating the application value of molecular markers in the study of plant genetic diversity [18,30,36]. The cpSSR markers developed in our study yielded reproducible polymorphic alleles in 203 samples from 16 *P. angulata* populations. This indicates that these cpSSR markers can be used as a reliable molecular tool for studying genetic diversity and population structure in *P. angulata*.

A total of 30 out of 54 cpSSR markers amplified electrophoretic bands with high levels of polymorphism, good stability, and high resolution in *P. angulata* populations, accounting for 55.56% of the newly developed markers. The polymorphic ratio of the cpSSR markers ranged from 25% to 100%, with an average of 81.29%, which was higher than the polymorphic ratios detected by SSR of 73.5% among *Dendrocalamus hamiltonii* [37], and 53.8% among celery cultivars [38]. The PIC values ranged between 0.550 and 0.945, with an average of 0.830, indicating that the cpSSR markers had good polymorphism and could be used to assess genetic diversity in *P. angulata* populations.

Genetic diversity is the sum of genetic information in a population, which is necessary for population persistence, adaptation, and evolution [36,39]. The interaction of drift, migration, mutation, and selection is the main factor causing genetic diversity in natural populations [14,40]. Using cpSSR markers, our study indicated that there is considerable genetic diversity among *P. angulate* populations (*Na* = 1.3161, *Ne* = 1.1754, *h* = 0.1023, and *I* = 0.1538). The proportion of polymorphism among *P. angulata* populations ranged from 6.41% to 54.49%, indicating that there were significant differences in polymorphic loci among populations. Furthermore, the XS population showed greater genetic diversity than the other 15 populations. Correspondingly, the complexity of genetic diversity was as follows: XS > YN > WZ > XJ > PJ > HG > JJ > NJZ > YW > TZ > LA > NH > LH > NJX > HZ > DQ. Compared to other populations, the DQ population from Deqing, Zhejiang Province had the lowest level of genetic diversity, with the smallest number of polymorphic loci, i.e., the lowest *Na* and *Ne* values. The reason for the lowest genetic diversity in the DQ population is probably because it contains a limited number of individuals adapted to specific habitats due to their isolation from other populations. Therefore, to more accurately evaluate the genetic diversity of this population in the future, more samples will be required. The average *h* and *I* values among the *P. angulata* populations were 0.1023 and 0.1538, respectively (<0.5), similar to those of *P. philadelphica* and *P. peruviana* [27,28,41,42]. The scattered and narrow distribution ranges, as well as small population sizes and large spatial distances between populations, limit pollination between populations, leading to self- and inbreeding. All of these factors may contribute to low genetic diversity.

Gene flow, negatively correlated with the genetic differentiation coefficient, is a basic microevolutionary phenomenon that often hinders genetic differentiation between populations and affects the maintenance of genetic diversity [14,43,44]. In our study, the level of gene flow among *P. angulata* populations was low (*Nm* = 0.2324), similar to that found in *P. philadelphica*, *P. peruviana*, and *M. savatieri* [28,41,45]. The main reasons for the low degree of genetic differentiation of among *P. angulata* populations may be related to the genetic mode and the dispersal distance of the seeds and pollen. The *Ht* value of *P. angulata* (0.3224) was higher than that of *P. philadelphica* (*Ht* = 0.292) [41]. The *Gst* value of 0.6827 for *P. angulata* indicates that 68.27% of the genetic variation occurred among populations, while 31.73% of the genetic variation occurred within populations. This is a similar result to that observed in *Rhodiola alsia* [46]. The AMOVA results also supported population differentiation, which indicated that the major genetic variance occurred among populations rather than within populations.

The population genetic structure reveals the distribution pattern of genetic diversity within and among populations [14,39,47]. Three methods, UPGMA and NJ clustering, PCoA, and Structure analysis, were combined to detect the genetic diversity and population structure in *P. angulata*. Many studies have reported a correlation between genetic distance and geographical location in the populations of certain plants [30,39,45,47]. Similar results were also obtained in our study. The UPGMA and NJ results both indicated that the six populations from the Zhejiang region (HZ, LA, LH, TZ, DQ, and YW) were jointly grouped into one cluster. These six populations were geographically close, especially the LH and TZ populations, and the genetic distance between them was also the smallest. The populations that were geographically distant from Zhejiang, such as the NJX, XJ, HG, and YN populations, were clustered together, while the JJ population from Jiangxi was clustered separately. These results indicated that the genetic distance between the *P. angulata* populations from these provinces and those from Zhejiang Province was relatively large, and the level of gene flow was very low. Interestingly, some populations did not show a clear correlation with geographical location. For example, the NJZ population from Jiangsu Province and four populations (XS, PJ, NH, and WZ) from Zhejiang Province were clustered together, although they were geographically distant. In our previous investigation of morphological phenotypic traits, we found that the plant height in these five populations was higher than that in the other populations. This might explain why they clustered together. Our study showed that the genetic diversity of *P. angulata* was not only related to geographical location but also likely associated with the geographical environment, human selection, and other factors. Furthermore, the PCoA and Structure analysis results were identical, and both supported the results of the UPGMA and NJ trees analyses. In a future study, more *P. angulata* population samples and more genome-wide molecular markers (such as SNPs, nSSRs, AFLPs, and others) will be used to more comprehensively evaluate the genetic diversity and structure of *P. angulata* populations.

## 4. Materials and Methods

### 4.1. Plant Materials and DNA Extraction

**Figure 7:**
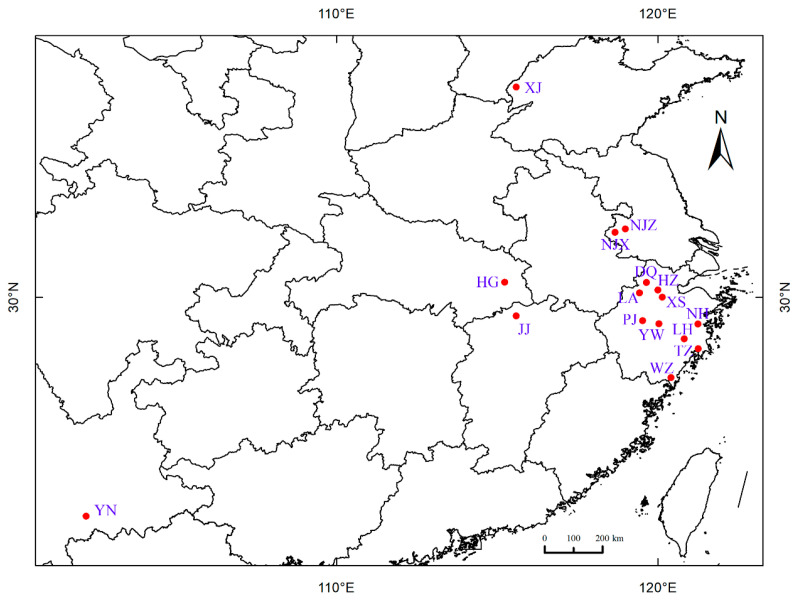
Sampling locations of the *P. angulata* used in this study.

**Figure 8:**
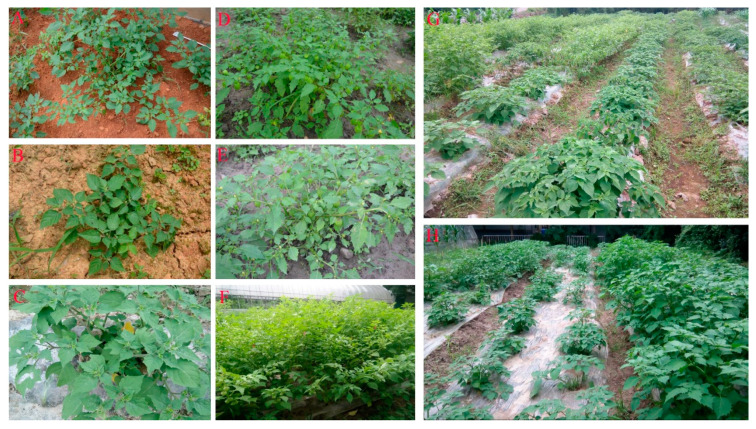
Partial germplasm resource sample phenotype and *P. angulata* planting resource nursery. (**A**) sample from XJ; (**B**) sample from JJ; (**C**) sample from HZ; (**D**) sample from XS; (**E**) sample from NH; (**F**) sample from WZ; (**G**,**H**) *P. angulata* planting resource nursery.

In total, 203 samples of wild *P. angulata* representing 16 populations were randomly collected from their main areas of distribution in China (Table 8, Figure 7). The identity of the samples was confirmed by reference to the species description provided in the herbarium at the Institute of Botany, Chinese Academy of Sciences, China (http://www.cvh.ac.cn/; accessed on 15 June 2021). The collected samples were planted during the regular crop growing season (early May) in 2018 at the field site of the Zhejiang Provincial Key Laboratory for Genetic Improvement and Quality Control of Medicinal Plants, Hangzhou Normal University, China. The field site was located at a geographic position of 30°32′18′′ N, 120°40′07′′ E and an altitude of 10 m. The phenotypes of some of the *P. angulata* materials are shown in Figure 8.

Fresh, young leaf tissues from three individuals of each sample were randomly collected for genomic DNA isolation. Genomic DNA was isolated from these samples as described in our previous studies [1,48]. The DNA quality was evaluated using 1.0% agarose gel electrophoresis, and the DNA quantity was determined using a UV spectrophotometer.

### 4.2. CpSSR Marker Development

In our earlier work, the complete chloroplast genome sequence of *P. angulata* was sequenced, annotated, and submitted to the National Center for Biotechnology Information (NCBI) GenBank database (GenBank accession no. MH045574) [49]. The SSR loci distributed throughout the *P. angulata* chloroplast genome were screened using MISA software (http://pgrc.ipk-gatersleben.de/misa/; accessed on 20 May 2022) [50]. The cpSSR motifs comprised 1–6 nucleotides containing the minimum number of repeats. Ten motifs contained mononucleotide repeats, five contained dinucleotide repeats, four contained trinucleotide repeats, and three each contained tetra-, penta-, and hexanucleotide repeats. Primer Premier 5 software was used to design cpSSR primers, and manual adjustments were made [51]. The parameters for designing the primers were set as follows: the primer length was 18–26 nucleotides, the annealing temperature was 55 °C ± 5 °C, and the amplification product size was 100–300 bp.

### 4.3. CpSSR Analysis

In total, 54 cpSSR primer pairs were selected and synthesized by Shanghai Sangon Biological Engineering Technology and Service Co. Ltd. (Shanghai, China). After a trial run of the 54 newly developed cpSSR primer pairs, 30 cpSSRs showing high definition, good stability, and abundant polymorphism were chosen for further analysis. cpSSR amplification was performed in 10 μL volumes including 1 μL of 10× buffer (200 mM Tris–HCl (pH 8.8), 100 mM KCl, 100 mM (NH_4_)_2_SO_4_, 20 mM MgCl_2_, and 1% TritonX-100), 0.8 μL of dNTPs (10 mmol/L), 1 µL each of forward and reverse primers (10 µM), 1 µL of Taq DNA polymerase (2 U/µL) (Beijing Dingguo Changsheng Biotechnology Co. Ltd., China), 50 ng of genomic DNA template, and 4.7 μL of ddH_2_O. The amplification program for the cpSSR-PCR was as follows: pre-denaturation at 94 °C for 5 min, followed by 32 cycles of denaturation at 94 °C for 50 s, annealing at 55–61 °C (depending on each primer’s T_m_ value) for 50 s, 72 °C for 1.5 min, and a final extension at 72 °C for 10 min. PCR products were separated on 8% nondenaturing-polyacrylamide gel (acrylamide: bisacrylamide 19:1) using *Trans2K* DNA markers (TransGen Biotech, Beijing, China) as size standards before being stained with silver.

### 4.4. Data Analysis

To ensure the accuracy of the results, each pair of primers was used for PCR amplification and electrophoresis detection twice, and only the cpSSR fragments with high definition and good stability were scored. Genetic variation at each locus was characterized in terms of the number of alleles. The PIC was calculated using the following formula: $PIC=1-\left(\sum_{i=1}^{n}p_{i}{}^{2}\right)-\left(\sum_{i=1}^{n-1}\sum_{j=i+1}^{n}2q_{i}{}^{2}q_{j}{}^{2}\right)$, where *n* is the number of alleles, *q_i_* is the frequency of the *i*-th allele, and *q_j_* is the frequency of the *j*-th allele [52]. The percentage of polymorphic loci (*PPL*), the number of observed alleles (*Na*), the number of effective loci (*Ne*), Shannon’s information diversity index (*I*), Nei’s genetic diversity index (*h*), and the total genetic variation of the population (*Ht*) were calculated using PopGene32 Version 1.32 (https://sites.ualberta.ca/~fyeh/popgene_download.html; accessed on 22 August 2022). The genetic variation within the population (*Hs*), population genetic differentiation coefficient (*Gst*), and gene flow (*Nm*) were also calculated using PopGene32 Version 1.32. On the basis of the genetic consistency of the populations, an unweighted group arithmetic mean (UPGMA) cluster diagram and three-dimensional principal coordinate analysis (PCoA), which enable the visualization of genetic variation distribution across populations, were constructed and carried out using NTSYS-PC 2.10e software [53]. On the basis of the genetic distance among the 203 individuals, a neighbor-joining (NJ) cluster diagram was constructed in MEGA X [54]. Analysis of molecular variance (AMOVA) and a two-dimensional principal coordinate analysis (PCoA) across individuals were computed using GenAlEx 6.5 software [55]. The population genetic structure was analyzed using the Bayesian clustering analysis method in STRUCTURE 2.3.4 software [56]. The estimated range of group K values was set to 2–15, and each K value was run 10 times. In addition, the length of burn-in period and MCMC (Markov chain Monte Carlo) parameters were set to 10,000 and 50,000, respectively. Lastly, using Structure Harvester online software (http://taylor0.biology.ucla.edu/structureHarvester; accessed on 22 August 2022), the results of the structure calculation were analyzed to find the best group K value [57,58].

## 5. Conclusions

In conclusion, this is the first study to develop a novel set of cpSSR markers and apply them to the investigation of genetic diversity and genetic structure in *P. angulata* populations. Our study revealed that most of the newly developed cpSSR markers had a high level of stability and polymorphism. The cpSSR analysis confirmed that *P. angulata* populations contain considerable genetic diversity, and there are high levels of genetic differentiation among populations. The 16 populations of *P. angulata* were clustered into four groups with some significant geography-related population structure and extensive admixture. Our study demonstrated that cpSSR markers can be used as a powerful tool for evaluating genetic diversity and population structure in *P. angulata*.

## Figures and Tables

**Table 1:** Characterization of cpSSRs in the chloroplast genome of *P. angulata*.

Parameter	Value
Total size of examined sequences (bp)	156,905
Total number of identified SSRs	57
Number of SSR containing sequences	1
Number of sequences containing more than 1 SSR	1
Number of SSRs present in compound formation	3
Repeat types	
Mononucleotide	39 (68.42%)
Dinucleotide	6 (10.53%)
Trinucleotide	5 (8.77%)
Tetranucleotide	7 (12.28%)

**Table 2:** Distributions of cpSSR motifs observed in the chloroplast genome of *P. angulata*.

SSR Motif	Number of Repeat Units in cp Genome								
Repeats	3	4	5	6	7	8	9	≥10	Total
Mononucleotide								39	39
(A)n	–	–	–	–	–	–	–	14	14
(C)n	–	–	–	–	–	–	–	1	1
(T)n	–	–	–	–	–	–	–	24	24
Dinucleotide			5		1				6
(AT)n	–	–	3			–	–	–	3
(TA)n	–	–	2		1	–	–	–	3
Trinucleotide		5							5
(AAG)n	–	1	–	–	–	–	–	–	1
(ACT)n	–	1	–	–	–	–	–	–	1
(TAA)n	–	1	–	–	–	–	–	–	1
(TTA)n	–	1	–	–	–	–	–	–	1
(TTC)n	–	1	–	–	–	–	–	–	1
Tetranucleotide	7								7
(AAAC)n	1	–	–	–	–	–	–	–	1
(CTAT)n	1	–	–	–	–	–	–	–	1
(CTTA)n	1	–	–	–	–	–	–	–	1
(TTTA)n	2	–	–	–	–	–	–	–	2
(TTTG)n	2	–	–	–	–	–	–	–	2
Total	7	5	5	0	1	0	0	39	57
Distribution frequency (%)	12.28	8.77	8.77	0	1.54	0	0	68.42	

**Table 3:** Statistical analyses of genetic polymorphisms in 30 cpSSR primer pairs.

Primer Name	Primer Sequences (5′–3′)	Repeat Motif	Tm (°C)	Sequence Size (bp)	No. of Alleles	Polymorphic Alleles	Polymorphic Rate (%)	PIC
KzcpSSR02	F: CGTAGAAAGACGAAAGTGGATT	(AAG)_4_	58	196	11	10	90.91	0.931
	R: AAACTCTTCGCTATTGGGTAAA							
KzcpSSR04	F: ATAGATAAATACACCAAACAACAAA	(T)_12_	58	231	12	11	91.67	0.945
	R: GATAGAAGTTAATCAGTAATGGGAA							
KzcpSSR17	F: TCAACAATGATCCACTAGACACT	(A)_20_	58	255	9	9	100.00	0.939
	R: TTTTCCCCTCAAATATGAATACT							
KzcpSSR18	F: AGGTTCTTTAAATTCCGTGG	(TTC)_4_	58	172	4	3	75.00	0.835
	R: TCCTTTTTCAAATCCTGCTG							
KzcpSSR19	F: TGATCGTGACCTTGAACCTGTT	(T)_13_	60	299	2	1	50.00	0.550
	R: TCCCTCTCTCTCCTTTTTTGCT							
KzcpSSR21	F: TTTTTCCTATTTTGACTTCTATG	(T)_13_	56	172	2	1	50.00	0.657
	R: ATCTGTCATTACGTGCGACTATC							
KzcpSSR23	F: ACAAGGAATGAAAAGAAAAAAGA	(CTTA)_3_	56	231	4	1	25.00	0.781
	R: AATAATAGATAGTAAATGGGTCG							
KzcpSSR24	F: GGGATGAATTGGATAAATATACAG	(TA)_5_	58	158	4	3	75.00	0.831
	R: TAGATGATTGATAATGTTCCTTTG							
KzcpSSR25	F: GAATAAAAAAAAGAATAGGGAA	(ACT)_4_	56	247	2	1	50.00	0.613
	R: TAGAATTGTGATAAATCGAAA							
KzcpSSR26	F: TTCTCCTGTTTCTCTTGTTTTTTT	(T)_11_	57	189	8	8	100.00	0.922
	R: CTCTTCTATTTGATTACTTGTTCT							
KzcpSSR29	F: CTTTGCGTTTTTCTTTCTTTT	(A)_11_	57	178	7	5	71.43	0.897
	R: ACCCAATTTTTATTTTTTACC							
KzcpSSR30	F: GAGTTTTTGACTTTCATTATTTTG	(T)_10_	58	188	4	4	100.00	0.862
	R: TTTTCTTCCCCGCATTTATC							
KzcpSSR31	F: AAAAGAAAAAGAAATCCATTTT	(A)_10_…(TTTG)_3_	57	176	4	1	25.00	0.780
	R: GTTGGGTTCATCCCTGTAGTAA							
KzcpSSR34	F: CTCTACAAGAAAATTGACCCCC	(A)_13_	58	187	6	5	83.33	0.865
	R: TGCTGAATCACAGACAAAAAAA							
KzcpSSR35	F: GATAAAGTCGGTTGATTAGGGT	(T)_16_	60	178	8	7	87.50	0.921
	R: ATTGAAAAATCGAAGAAAAGCC							
KzcpSSR36	F: CCCATTACCATTTCTTTTTGT	(T)_10_	60	179	4	4	100.00	0.858
	R: TGAAGTATCCAGGCTCCGTTT							
KzcpSSR37	F: TTTGTTTTGTAATGGATAGTTGC	(T)_12_	56	251	2	2	100.00	0.714
	R: TTTTTGTTATTGGGATAGGTGAA							
KzcpSSR38	F: TCTTTGTTTTGTAATGGATAGTTG	(AT)_5_	60	256	2	2	100.00	0.719
	R: GTTTTTTTGTTATTGGGATAGGTG							
KzcpSSR39	F: TTGGCTGTTATTCAAAAGGTC	(T)_11_	58	188	5	3	60.00	0.836
	R: ACAATCAACATACGGTTCCTT							
KzcpSSR41	F: TTCTTATTTAATGGTTAGGTCCG	(T)_10_	60	175	4	4	100.00	0.854
	R: AAAGCATCAATACGCATTCATAC							
KzcpSSR42	F: ATTGTGGGTATAATGGTAGATGC	(T)_16_	58	271	7	6	85.71	0.896
	R: TGGAAGAAGAAGTAGAAAAAGGA							
KzcpSSR44	F: AAAATGGAAAGTTCGACACAA	(A)_12_	58	143	3	2	66.67	0.772
	R: GAAGAGAAGCAAATGAAAGGC							
KzcpSSR45	F: GTGACGATACTGTAGGGGAGG	(A)_12_	58	239	7	5	71.43	0.904
	R: ATTTCGGGTTAAGAAGATGTG							
KzcpSSR46	F: AGGTCGTGTCATCTTTCTTCCAT	(A)_10_	60	153	5	4	80.00	0.850
	R: CACAAAACCCCTTTCTACTCAAT							
KzcpSSR47	F: GGAAAGAAACAAAAAAAAGAAA	(A)_11_	58	125	4	4	100.00	0.857
	R: TGAGAAAGGAGAATAGGAATGA							
KzcpSSR48	F: CAATTTTCAGATTCAGTTTGACTA	(T)_13_	59	199	5	5	100.00	0.885
	R: AAGAAACCAAAGAATGGCTTATCA							
KzcpSSR49	F: GCCATTCTTTGGTTTCTTTT	(T)_10_	56	160	4	4	100.00	0.861
	R: TCCTTTTTTGAGCCCATTTT							
KzcpSSR50	F: ATCAATGAAGGTAATAGAATA	(T)_10_	55	179	6	6	100.00	0.903
	R: CAAACAAAAAGAGAAGAGAAA							
KzcpSSR51	F: CGAGGTGTGAAGTGGGAGAGA	(T)_12_	60	145	9	9	100.00	0.935
	R: CGACGCCAGGATGATAAAAAG							
KzcpSSR53	F: GTAATTTCATAGAGTCATTCGGTC	(AT)_5_	60	236	2	2	100.00	0.716
	R: CCAAACTGTACAAGCTTCTTCCAA							
Average					5.2	4.4	81.29	0.830
Total					156	132		

**Table 4:** Genetic diversity analysis of 16 populations of *P. angulata*.

Population	*Na*	*Ne*	*h*	*I*	*PL*	*PPL* (%)
HZ	1.1154 ± 0.3205	1.0879 ± 0.2623	0.0476 ± 0.1389	0.0685 ± 0.1972	18	11.54
XS	1.5449 ± 0.4996	1.2984 ± 0.3705	0.1743 ± 0.1974	0.2640 ± 0.2798	85	54.49
LA	1.2051 ± 0.4051	1.0873 ± 0.2268	0.0536 ± 0.1295	0.0839 ± 0.1909	32	20.51
DQ	1.0641 ± 0.2457	1.0371 ± 0.1663	0.0215 ± 0.0902	0.0325 ± 0.1316	10	6.41
YW	1.3077 ± 0.4630	1.1848 ± 0.3122	0.1093 ± 0.1792	0.1628 ± 0.2609	48	30.77
PJ	1.4551 ± 0.4996	1.2226 ± 0.3325	0.1324 ± 0.1850	0.2020 ± 0.2660	71	45.51
NH	1.1795 ± 0.3850	1.1368 ± 0.3120	0.0749 ± 0.1672	0.1079 ± 0.2381	28	17.95
LH	1.1538 ± 0.3620	1.0969 ± 0.2576	0.0555 ± 0.1418	0.0821 ± 0.2048	24	15.38
TZ	1.2244 ± 0.4185	1.1689 ± 0.3255	0.0951 ± 0.1796	0.1376 ± 0.2584	35	22.44
WZ	1.5000 ± 0.5016	1.2375 ± 0.3327	0.1428 ± 0.1852	0.2195 ± 0.2656	78	50.00
NJZ	1.3205 ± 0.4682	1.1466 ± 0.2730	0.0902 ± 0.1600	0.1390 ± 0.2351	50	32.05
NJX	1.1346 ± 0.3424	1.0842 ± 0.2321	0.0498 ± 0.1321	0.0741 ± 0.1938	21	13.46
JJ	1.3910 ± 0.4896	1.2323 ± 0.3655	0.1323 ± 0.1923	0.1982 ± 0.2739	61	39.10
HG	1.4231 ± 0.4956	1.2306 ± 0.3458	0.1344 ± 0.1909	0.2019 ± 0.2738	66	42.31
XJ	1.4936 ± 0.5016	1.2692 ± 0.3672	0.1559 ± 0.1975	0.2345 ± 0.2809	77	49.36
YN	1.5449 ± 0.4996	1.2858 ± 0.3634	0.1673 ± 0.1974	0.2529 ± 0.2808	85	54.49
Average	1.3161 ± 0.4311	1.1754 ± 0.3028	0.1023 ± 0.1665	0.1538 ± 0.2395	49	31.61

**Table 5:** Nei’s genetic identity (above diagonal) and genetic distance (below diagonal).

Population	HZ	XS	LA	DQ	YW	PJ	NH	LH	TZ	WZ	NJZ	NJX	JJ	HG	XJ	YN
HZ		0.5009	0.9221	0.8996	0.9238	0.5339	0.4446	0.9237	0.8993	0.5293	0.4860	0.8845	0.8438	0.8892	0.9015	0.8935
XS	0.6914		0.5267	0.4994	0.5356	0.9211	0.9171	0.5046	0.5266	0.9075	0.9093	0.5160	0.6482	0.5920	0.5975	0.5809
LA	0.0811	0.6411		0.9419	0.9479	0.5570	0.4662	0.9507	0.9426	0.5143	0.4897	0.9054	0.8538	0.8998	0.9053	0.9119
DQ	0.1059	0.6943	0.0599		0.9612	0.5199	0.4403	0.9146	0.9197	0.4858	0.4409	0.8930	0.8254	0.8881	0.8740	0.8716
YW	0.0793	0.6243	0.0535	0.0396		0.5493	0.4727	0.9392	0.9346	0.5237	0.4852	0.9150	0.8505	0.9183	0.9115	0.9124
PJ	0.6275	0.0822	0.5853	0.6541	0.5991		0.9337	0.5370	0.5433	0.9080	0.9106	0.5101	0.6774	0.5933	0.6154	0.5939
NH	0.8106	0.0865	0.7631	0.8202	0.7492	0.0686		0.4603	0.4658	0.8900	0.9171	0.4374	0.5835	0.5175	0.5362	0.5092
LH	0.0794	0.6840	0.0505	0.0893	0.0627	0.6217	0.7758		0.9677	0.5038	0.4833	0.9263	0.8549	0.9179	0.9302	0.9204
TZ	0.1062	0.6414	0.0591	0.0837	0.0677	0.6100	0.7640	0.0329		0.5443	0.4970	0.9167	0.8608	0.9228	0.9217	0.9194
WZ	0.6361	0.0971	0.6649	0.7220	0.6469	0.0966	0.1165	0.6856	0.6083		0.9346	0.5065	0.6657	0.6034	0.6098	0.5979
NJZ	0.7216	0.0951	0.7139	0.8190	0.7232	0.0937	0.0865	0.7271	0.6991	0.0676		0.4896	0.6462	0.5794	0.5964	0.5650
NJX	0.1227	0.6617	0.0994	0.1131	0.0888	0.6731	0.8268	0.0765	0.0870	0.6802	0.7141		0.8492	0.9374	0.9413	0.9336
JJ	0.1698	0.4335	0.1581	0.1919	0.1619	0.3896	0.5388	0.1567	0.1499	0.4070	0.4366	0.1634		0.9012	0.9021	0.8985
HG	0.1174	0.5243	0.1056	0.1187	0.0852	0.5221	0.6587	0.0857	0.0804	0.5051	0.5457	0.0647	0.1041		0.9534	0.9551
XJ	0.1037	0.5150	0.0995	0.1347	0.0927	0.4855	0.6233	0.0723	0.0815	0.4947	0.5168	0.0604	0.1031	0.0477		0.9513
YN	0.1126	0.5432	0.0922	0.1375	0.0917	0.5210	0.6750	0.0829	0.0841	0.5143	0.5710	0.0687	0.1071	0.0460	0.0499	

**Table 6:** Genetic variation in the 16 populations of *P. angulata* based on cpSSR markers.

Total Gene Diversity (*Ht*)	Population Genetic Diversity (*Hs*)	Coefficient of Genetic Differentiation (*Gst*)	Gene Flow (*Nm*)
0.3224 ± 0.0284	0.1023 ± 0.0064	0.6827	0.2324

**Table 7:** Summary of the AMOVA results for 203 samples of *P. angulata*.

Source	df	SS	MS	Est. Var.	Percentage (%)	PhiPT
Among pops	15	10,086.383	672.426	51.470	71	0.710 (*p* = 0.001)
Within pops	187	3923.824	20.983	20.983	29	
Total	202	14,010.207		72.453	100	

Note: df, degree of freedom; SS, sum of squared observations; MS, mean of squared observations. PhiPT = AP/(WP + AP) = AP/TOT; Key: AP = estimated variance among populations, WP = estimated variance within populations.

**Table 8:** Sample sizes and location information for 16 populations of *P. angulata*.

Number	Population Code	Sample Size	Locations
1	HZ	10	Jianggan, Hangzhou, Zhejiang, China
2	XS	14	Xiaoshan, Hangzhou, Zhejiang, China
3	LA	13	Tianmushan, Lin’an, Zhejiang, China
4	DQ	6	Deqing, Huzhou, Zhejiang, China
5	YW	12	Yiwu, Jinhua, Zhejiang, China
6	PJ	14	Pujiang, Jinhua, Zhejiang, China
7	NH	10	Nihai, Ningbo, Zhejiang, China
8	LH	12	Linhai, Taizhou, Zhejinag, China
9	TZ	15	Jiaojiang, Taizhou, Zhejiang, China
10	WZ	12	Wenling, Wenzhou, Zhejiang, China
11	NJZ	15	Xixia, Nanjing, Jiangsu, China
12	NJX	12	Xuanwu, Nanjing, Jiangsu, China
13	JJ	13	Xunyang, Jiujiang, Jiangxi, China
14	HG	15	Luotian, Huanggang, Hubei, China
15	XJ	14	Xiajin, Dezhou, Shangdong, China
16	YN	16	Baohua, Honghe, Yunnan, China

## Data Availability

All data are included in the manuscript.

## Supplementary Materials

The following supporting information can be downloaded at https://www.mdpi.com/article/10.3390/plants12091755/s1: Table S1. Information on 57 SSR motifs detected in the chloroplast genome of *P. angulata*; Table S2. Information on the 54 cpSSR primer pairs developed in this study.
